# Oligodendrocyte precursor cells: the multitaskers in the brain

**DOI:** 10.1007/s00424-023-02837-5

**Published:** 2023-07-04

**Authors:** Li-Pao Fang, Xianshu Bai

**Affiliations:** Molecular Physiology, Center for Integrative Physiology and Molecular Medicine, University of Saarland, 66421 Homburg, Germany

**Keywords:** Oligodendrocyte precursor cells, OPC, Neural circuits, Neuron-OPC interaction, Immunomodulator, Blood-brain barrier

## Abstract

In the central nervous system, oligodendrocyte precursor cells (OPCs) are recognized as the progenitors responsible for the generation of oligodendrocytes, which play a critical role in myelination. Extensive research has shed light on the mechanisms underlying OPC proliferation and differentiation into mature myelin-forming oligodendrocytes. However, recent advances in the field have revealed that OPCs have multiple functions beyond their role as progenitors, exerting control over neural circuits and brain function through distinct pathways. This review aims to provide a comprehensive understanding of OPCs by first introducing their well-established features. Subsequently, we delve into the emerging roles of OPCs in modulating brain function in both healthy and diseased states. Unraveling the cellular and molecular mechanisms by which OPCs influence brain function holds great promise for identifying novel therapeutic targets for central nervous system diseases.

## Introduction

Proper brain function is achieved through coordinated activity between neurons and glial cells. Glial cells control the spatio-temporal pattern of neural circuits by regulating cell density, synaptic activity, and the conduction velocity of action potentials. While the contribution of microglia and astrocytes to neural synaptic plasticity has been extensively studied, the role of oligodendrocyte precursor cells (OPCs) in neuronal network activity is just beginning to be elucidated.

**Fig. 1:**
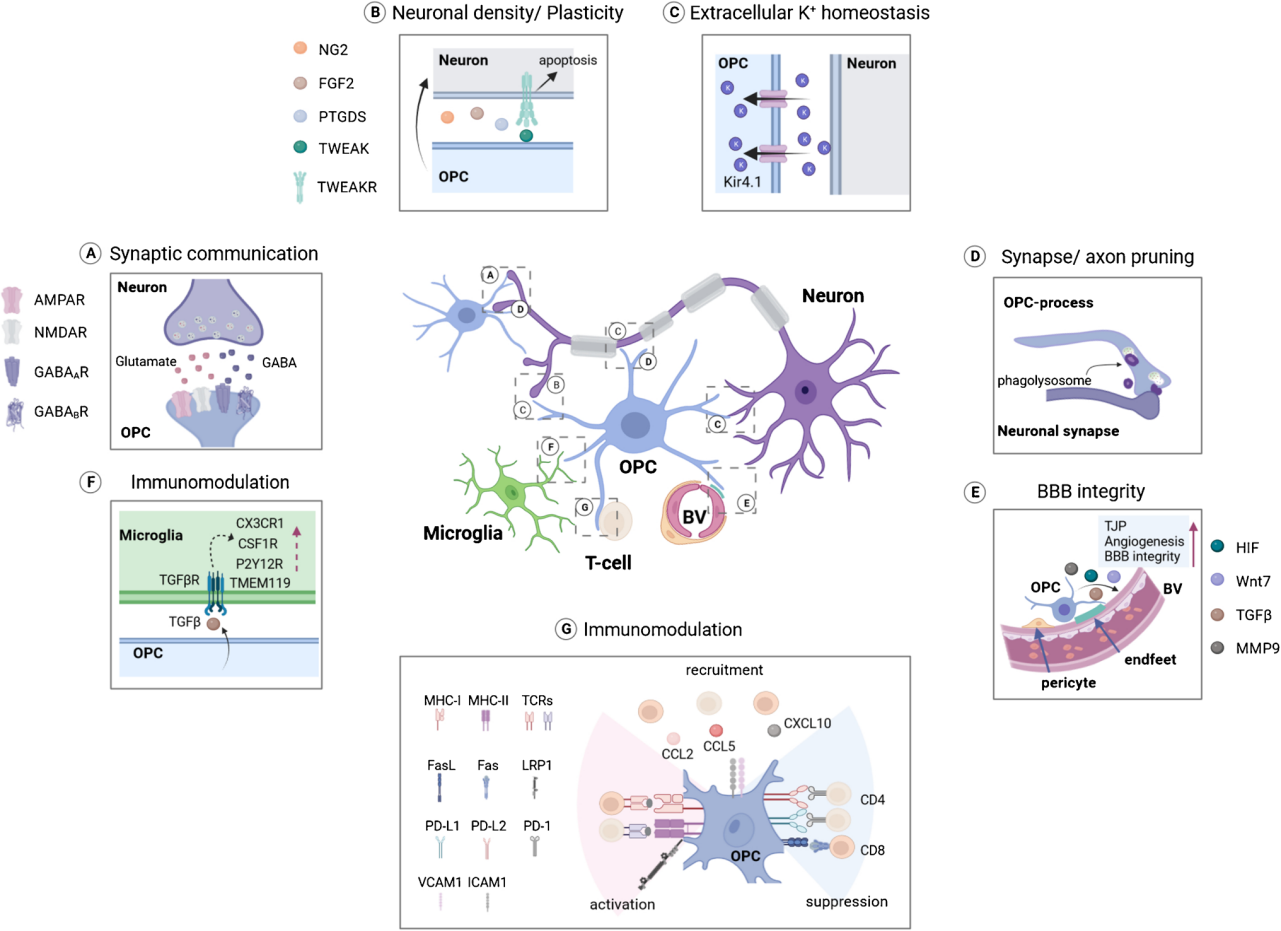
Integration of OPCs into neural circuits occurs through multiple pathways. **A** OPCs receive glutamatergic signals via AMPA and NMDA receptors, as well as GABAergic input through GABA_A_ and GABA_B_ receptors expressed on their postsynaptic membrane. **B** OPCs regulate neuronal density, activity, and synaptic plasticity by releasing TNF-related weak inducer of apoptosis (TWEAK), fibroblast growth factor 2 (FGF2), and prostaglandin D2 synthase (PTGDS), and cleaved ectodomain of NG2 protein. **C** OPCs sense neuronal activity through the potassium channel Kir4.1. Upon axonal stimulation, Kir4.1 mediates an increase in intracellular potassium concentration in OPCs, potentially contributing to the maintenance of extracellular potassium homeostasis. **D** In the juvenile brain, OPCs phagocytose axons and excitatory presynapses, thus mediating neural network activity. **E** The interaction of OPCs with components of the neural vascular unit, including endothelial cells, pericytes, and astroglial endfeet, is crucial for maintaining the integrity of blood-brain barrier. For example, OPCs release hypoxia-inducible factor (HIF) to promote angiogenesis and tumor growth factor β (TGFβ) to increase tight junction protein (TJP) expression and subsequent BBB integrity. **F** OPCs modulate microglia activity and immune response by releasing TGFβ, which acts on the TGFβ receptor (TGFβR) of microglia. Activation of TGFβR may upregulate proteins such as CX3CR1, CSF1R, P2Y12R, and TMEM119, since knockdown of TGFβR in microglia led to downregulation of these proteins. **G** OPCs also recruit T cells by releasing chemokines, such as C-C motif ligand 2 (CCL2), CCL5, and CXCL10. The expression of major histocompatibility complex I and II (MHC-I/II) by OPCs activates T cells, while the expression of programmed death ligands (PD-L) suppresses T cell activity. Furthermore, OPCs can induce T cell apoptosis through Fas ligand (FasL). (created with BioRender.com)

As their name suggests, OPCs generate oligodendrocytes (OLs), the only myelin-forming cells in the central nervous system (CNS), throughout life [73]. OPCs maintain a relatively stable cell density, constituting approximately 5–8% of the total cell population in the CNS, as they continuously self-renew [7, 32]. Despite their ubiquitous distribution in the parenchyma, OPCs represent a rather heterogeneous population in respect to their origin [28, 40], location [39, 40, 64, 76], and receptor/channel expression [7, 41,  67]. In addition, OPCs exhibit distinct morphology in different brain regions. Their processes in gray matter tend to be radially oriented, whereas in white matter they exhibit a more elongated shape [32]. Nevertheless, in both gray and white matter, their processes consist of lamellipodia and filopodia [32, 51], and many of them are in close contact with parasynaptic areas and nodes of Ranvier [12, 13, 32, 48, 63] (Fig. 1). This physical contact implies direct communication between OPCs and neurons. Indeed, in 2000, a direct synaptic neurotransmission on OPCs was first identified in the rat hippocampus [9]. Pyramidal neurons release glutamate at the synaptic cleft, and OPCs integrate this signal with the α-amino-3-hydroxy-5-methyl-4-isoxazolepropionic acid (AMPA) receptor expressed on the postsynaptic membrane [9]. Subsequently, synaptic communication mediated by N-methyl D-aspartate (NMDA) receptor and gamma-aminobutyric acid (GABA) A receptors of OPCs has been observed in different brain regions and at different ages of mice and rats [27, 35, 53] (Fig. 1A). Both excitatory and inhibitory synaptic neurotransmission are involved in the OPC proliferation, differentiation, and subsequent myelination, which have been extensively reviewed by others [5, 24, 36]. Recently, a large body of evidence is mounting that OPCs are heterogeneously involved in the communication with neurons and other cells in the CNS. In this review, we elaborate on the functional heterogeneity of OPCs, focusing on their direct and indirect impact on neural circuits and brain functions in health and disease.

## Integration of OPCs into local neural circuits

OPCs are also known as NG2 glia as they express NG2 protein (also known as chondroitin sulfate proteoglycan 4). The expression of the membrane-spanning NG2 protein makes OPCs a distinct contributor to synaptic plasticity. Sakry et al. have reported that the ectodomain of NG2 protein, cleaved by ADAM10 (A disintegrin and metalloproteinase domain-containing protein 10), is involved in AMPA receptor-mediated synaptic neurotransmission in cortical pyramidal neurons (Fig. 1B). Pharmacological inhibition of NG2 cleavage reduced NMDA receptor-dependent long-term potentiation (LTP), suggesting a critical function of the ectodomain NG2 protein in synaptic plasticity [57]. Notably, the intracellular domain of NG2 also regulates the expression of neuromodulators [58]. Overexpression of the intracellular domain of the NG2 protein in Oli-neu cells (a well-established OPC cell line [26]) increased the expression of prostaglandin D2 synthase (PTGDS) (Fig. 1B), which regulates prostaglandin D_2_ (PGD_2_) levels in the CNS by catalyzing the conversion of prostaglandin H_2_ to PGD_2_. PGD_2_ is a potential neuromodulator involved in human sleep and emotion, as well as in the regulation of neural circuits in inflammation [1, 59, 65]. The modulation of OPCs in neural circuits is more than just the expression of NG2 protein. Birey et al. attempted a rather thorough approach to ablate all OPCs in the mouse brain and found that these mice exhibited depressive-like behavior [10]. These mutant mice exhibited impaired glutamatergic signaling in the prefrontal cortex (PFC), as the amplitude of miniature excitatory postsynaptic currents (mEPSCs) from pyramidal neurons in the PFC was significantly reduced. This may be due to the loss of OPC-derived fibroblast growth factor 2 (FGF2), as knockdown of FGF2 in OPCs caused similar depressive-like behaviors in mice [10] (Fig. 1B). All these studies highlight the NG2 protein and its cellular source, OPCs, as key regulators of the excitatory neural network.

Recently, two independent studies have demonstrated the significance of OPCs in cortical inhibition. Specific deletion of the B1 subunit of the GABA B receptor in OPCs at the postnatal day 7 and 8 resulted in the survival of supernumerary interneurons in the adult medial prefrontal cortex (mPFC) [22]. However, the amplitude of spontaneous inhibitory postsynaptic current (sIPSC) from layer V pyramidal neurons as well as the vGAT density was reduced in the mutant mouse mPFC. All of these changes led to a reduction in the cortical inhibitory tone in the adult brain and impaired cognition in the mice. Early postnatal disruption of GABA A receptor-mediated neurotransmission in OPCs caused a similar reduction in inhibitory tone in the somatosensory cortex [8]. The GABA A receptor γ2 subunit is specifically involved in synaptic communication between interneurons and OPCs during the first postnatal weeks [6]. Genetic deletion of the γ2 subunit selectively in OPCs from the postnatal day 3 resulted in impaired E/I balance in the somatosensory cortex [8]. In the somatosensory cortex of these mice, parvalbumin-expressing interneurons displayed suppressed activity, reduced myelination, and an imbalance between excitation and inhibition. Concomitantly, these mice were unable to perform whisker-dependent texture discrimination, suggesting a dysfunctional cortical sensory circuit in these mutant mice. These two studies highlight the importance of GABAergic neurotransmission on OPCs in fine-tuning of neural circuits. Of note, OPCs can also form synaptic complexes with hippocampal interneurons. OPCs release GABA via synaptobrevin 2/vesicle-associated membrane protein 2 (VAMP2), which acts on proximal interneurons to enhance the inhibitory synaptic neurotransmission [86]. All these studies suggest that OPCs, by forming postsynapses and/or potential presynapses with neurons, or even by physical contact, modulate interneuron activity and inhibitory circuits in the brain.

Notably, by activating a Wnt/β-catenin signaling pathway, OPCs regulate both inhibitory and excitatory synapse formation. When disrupted-in-schizophrenia-1-Δ3 (DISC1-Δ3), a major DISC1 variant lacking exon 3, was overexpressed in OPCs, the mutant mice exhibited schizophrenia-like behavior [82]. In addition, synaptogenesis was suppressed in the PFC of these mutant mice. This disruption was attributed to the hyperactive Wnt/β-catenin pathway in OPCs, which subsequently upregulates Wnt inhibitory factor 1 (Wif1). Inhibition of Wif1 in OPCs could rescue the synapse loss and behavioral deficits of mutant mice, suggesting that OPCs are engaged in synaptogenesis via the Wnt signaling pathway [82]. The contribution of OPCs in psychological disorders is also suggested by post-mortem brain samples with a history of child abuse. In the PFC of these victims, the density and morphological complexity of the perineuronal net (PNN) was increased [71]. Single-nucleus transcriptomic and immunohistological analysis further showed that the canonical component of the PNN was enriched in OPCs and upregulated in the samples from child abuse victims. This implicates that OPC-mediated PNN formation is involved in impaired neuroplasticity of cortical circuits induced by early-life adversity. The impact of early-life stress on OPCs goes further. Parental isolation during the first two postnatal weeks reduced the number of hippocampal OPCs. This alteration led to maldevelopment of the astrocyte network and subsequently impaired neuronal activity and psychiatric behavior [79].

Oligodendrocyte transcription factor 2 (Olig2) is essential for OPC differentiation and has long been considered to be ubiquitously expressed in OPCs. However, a small population of OPCs was found to be immunonegative for Olig2 in the mouse brain [21]. The emergence of this population of cells appeared to be temporal and correlated with the (re-)establishment of the neural network. This population was enriched in the juvenile brain and almost disappeared with age. Acute brain injury and complex motor learning triggered the re-emergence of these cells in the associated brain regions, i.e., ipsilateral cortex of acute brain injury or hippocampus after motor learning [21]. Olig2 recruits SETDB1 to modify H3K9me3 of the Sox11 gene, which is an inhibitory factor for OPC differentiation [84]. Therefore, it is plausible that OPCs switch their fate commitment and remain in the precursor stage to modify the neural circuits as required by the establishment of neural network. Further investigation showed that these Olig2-negative OPCs were derived from Olig2-positive OPCs, but were less proliferative. Similarly, a cluster of so-called “quiescent” OPCs was observed in the zebra fish spinal cord [39]. These “quiescent” OPCs, located in the neuronal soma-enriched area, did not express the mitotic marker Ki67 and rarely differentiated into oligodendrocytes. Rather, these cells generate another subset of OPCs that are preferentially positioned in the axon-dendrite-enriched region, with higher motility and differentiation rate [39]. Thus, all these studies suggest that OPCs integrate into neural circuits heterogeneously at the micromilieu.

Proper brain function is highly dependent on an extremely exquisite microenvironment. For example, the local extracellular K^+^ concentration (K^+^_e_) is critical for the membrane potential and excitability of neurons. There is a transient increase in K^+^_e_ concentration during the repolarization phase of an action potential. Rapid recovery of the K^+^_e_ concentration is therefore extremely important for maintaining a precise chemical K^+^ gradient between the intra- and extracellular membrane and the resting potential. K^+^_e_ can be regulated by potassium channels expressed in astrocytes [77], microglia [56], and OPCs. The “buffering” of K^+^_e_ by OPCs is mainly achieved by inwardly rectifying K^+^ (Kir) channels [38], in particular Kir4.1 (encoded by *Kcnj10* gene) (Fig. 1C). Stimulation of axons induced a slow inward current of K^+^ in nearby OPCs. Such a current could be abolished in the presence of the Kir blocker Ba^2+^ in the bath or when the cells were recorded with CsCl-based intracellular solution or acidic intracellular solution, conferring to OPCs the sensor of extracellular K^+^ with their Kir4.1 channels (Fig. 1C). A further characterization of Kir4.1 function in OPCs was very recently performed using NG2-CreER^T2^ x Kcnj10^fl/fl^ mice [72]. In the mutant mouse CA1 region, the theta burst-stimulated long-term potentiation (LTP) was impaired, suggesting a critical contribution of OPCs to hippocampal synaptic plasticity. In addition, OPCs from mutant mice exhibited much larger and longer spontaneous and evoked postsynaptic currents (PSCs), indicating that deletion of Kir4.1 channels enhances synaptic input to OPCs, which could induce rapid calcium activity in OPCs, potentially through voltage-gated calcium channels [70]. Interestingly, deletion of Kir4.1 channels in the OL lineage cells using Olig2-Cre x Kcnj10^fl/fl^ mice induced a slight upregulation of the L-type voltage-gated Ca^2+^ channel Cav1.2 in OPCs [30]. Taken together, these observations suggest a potential compensatory role of Cav1.2 in the rapid recovery of neuronal membrane potential. Although it is unknown whether Cav1.2 is involved in the regulation of neuronal activity by OPCs, conditional deletion of Cav1.2 and Cav1.3 in OPCs impaired the long-term potentiation (LTP) and NMDA induced long-term depression (LTD) in the hippocampus [87]. These studies suggest that OPCs may modulate the long-term neural circuit plasticity and synaptic function via Cav1.2 and Cav1.3 channels.

Taken together, OPCs regulate neural circuits by modulating neuronal activity and synapse formation in health and disease.

## Synapse pruning and axonal remodeling by OPCs

In addition to synaptogenesis, OPCs also participate in axonal remodeling at different stages of development. In the zebra fish optic tectum, the axons of retinal ganglion cells (RGC) arrive at the tectum at 2 days post fertilization (dpf). When OPCs were ablated from two dpf, RGC axons developed abnormal branching as well as enlarged axon arbors [81]. However, late ablation of OPCs from seven dpf induced aberrant axonal remodeling, i.e., decreased axonal elimination but increased axon additions, resulting in impaired visual processing. This study suggests that OPC-mediated axonal remodeling differs at different developmental stages. This may be due, at least partially, to the nature of the heterogeneity of OPCs, which are generated in different waves during the development. In the mouse brain, OPCs are generated in three waves (discussed in more detail in the next section). Remarkably, the majority of the first two waves are eliminated during the first two postnatal weeks. This process slightly precedes the elimination phase of synapses that occurs during the second and third postnatal weeks [33], indicating that the third wave of OPCs generated during the perinatal days are more likely to be engaged in synapse pruning. Recently, Buchanan et al. visualized the axonal fragments in proximal OPC processes in the cortex of p36 mice using high-throughput transmission electron microscopy [11] (Fig. 1D). Further single-nucleus RNA sequencing analysis showed that numerous phagolysosome genes were abundantly expressed in the cortical OPCs as well as oligodendrocytes at the postnatal day 56, suggesting that OPCs may participate in synapse pruning. In parallel, another independent study showed that OPCs engulf thalamocortical presynapses in the visual cortex as early as postnatal day 10 [4]. These results may also explain the findings of Xiao et al. that early OPC ablation reduced axonal elimination. In addition, when microglia were depleted pharmacologically with a colony stimulating factor 1 receptor (CSF1R) inhibitor, the volume of vGlut in OPC processes was reduced [4]. This may be due to a crosstalk between microglia and OPCs regulating OPC-mediated pruning. However, further studies are required to address such interactions for synaptic pruning during neural network formation.

## OPCs regulate interneuron migration and apoptosis

In the mouse brain, OPCs are born in three successive waves: the first wave from the Nkx2.1-expressing precursors in the medial ganglionic eminence (MGE) and the embryonic preoptic area (ePOA) at embryonic day (E) 11.5–12.5, the second wave from the Gsx2-expressing precursors in the lateral and medial ganglionic eminences at E14.5, and from the Emx1^+^ cells in the cortex during perinatal days [28]. Interestingly, the majority of GABAergic interneurons are generated around E11.5–12.5 from the same precursors in the MGE and ePOA as the first wave of OPCs [34]. Although OPCs share the same progenitors and a similar birth time with interneurons, they exclude interneurons from the blood vessels (BV) during migration [31]. This repulsion is mediated by Sema6a/6b, which is expressed on OPCs and binds to the Plxna3 receptor in interneurons, thereby achieving unidirectional contact repulsion. This seemingly competing mechanism is actually essential for the penetration and correct colonization of interneurons in the brain, as the depletion of the first wave of OPCs disrupted interneuron migration and distribution in the cortex. Once established in the cortex, interneurons connect with target cells. Only those cells that receive retro-trophic signals from the target cells can survive, while the rest undergo programmed cell death during the first two postnatal weeks [34, 66]. At a similar time, the first wave of OPCs in the dorsal cortex is also largely eliminated with an unknown mechanism [28]. However, the surviving OPCs are found to preferentially form synaptic connections with interneurons of the same origin [52]. Interestingly, when the death of both OPCs and interneurons were intervened by deleting Bax genes in the cells derived from Nkx2.1^+^ progenitors using Nkx2.1 x Bax^fl/fl^ mice, this preferential connectivity was reduced [52]. In addition, the cortical E/I ratio was reduced in these mice, suggesting that the correct removal of the first-wave OPCs and interneurons is pivotal for proper brain function. Interestingly, OPCs also attribute to the developmental loss of interneurons during the first two postnatal weeks [22]. Conditional deletion of GABA_B_R in OPCs at the first postnatal week, just after the generation of the third-wave OPCs, attenuated interneuron apoptosis, and subsequently, more interneurons were found in the adult mouse mPFC. Further mechanistic studies revealed that OPCs release tumor necrosis factor (TNF)-related weak inducer of apoptosis (TWEAK or APO3L) upon GABA B receptor activation (Fig. 1B). TWEAK release from OPCs may be rather local at the contact site where the TWEAK receptors of interneurons are recruited [15], thereby inducing specific interneuron apoptosis [22] (Fig. 1B). It is not clear whether TWEAK is released by the first and/or the third-wave OPCs. Since the first-wave OPCs assist interneuron migration, the third-wave OPCs are more likely to execute interneuron elimination. Taken together, it is possible that the first-wave OPCs are born to associate interneuron function in the cortex. During the embryonic stage, first-wave OPCs guide the migration of interneurons to their destination. After birth, although the majority of these OPCs are eliminated, the survived first-wave OPCs form synaptic connection with interneurons [52]. However, the newborn third-wave OPCs exert a distinct function by optimizing interneuron density and synapse pruning (as mentioned above) [4, 11, 22]. Indeed, single cell transcriptomic studies suggested that embryonic OPCs and postnatal OPCs express a distinct gene profile [40]. Furthermore, it is even possible that the third-wave OPCs are involved in the phasing-out of the first-wave OPCs. However, further studies are needed to address these hypotheses.

## OPCs for blood-brain barrier integrity

The blood-brain barrier (BBB) is crucial for maintaining brain homeostasis through a highly selective exchange of substances between the brain parenchyma and the blood [18]. The BBB also acts as a “shield” to prevent pathogenic influences in the circulating blood from entering the brain parenchyma. The BBB is composed of endothelial cells that form the blood vessels (BV), while its integrity is highly dependent on regulation by pericytes, astroglial endfeet, and OPCs [18]. Embryonic vasculogenesis is controlled by region-specific transcription factors such as Nkx2.1. As mentioned above, the first wave of OPCs originates from Nkx2.1^+^ progenitors and migrates dorsally along the BVs [31, 74]. Nkx2.1^+^ OPCs are found either on the sprouting endothelial tip cells or adhering to the vessel walls. Such intense physical contact is not only required for OPC migration, but also for vessel formation during development. Conditional deletion of Nkx2.1^+^ progenitor-derived OPCs in Nkx2.1-Cre x Rosa-DTA or NG2-Cre x Rosa-DTA transgenic mice reduced the density and branching of BVs at E18.5 [43]. However, one should note that neither of these mouse lines is specific for Nkx2.1-derived OPCs. The Nkx2.1-Cre line not only targets OPCs, but also interneurons. As discussed above, OPCs and interneurons interact during developmental migration. Similarly, in NG2-Cre x Rosa-DTA mice, pericytes are also ablated as they also express NG2 protein. Pericytes are indispensable for BV formation and BBB function [18, 19]. Therefore, the atypical angiogenesis in these mice cannot be seen solely as a consequence of OPC loss. The contribution of OPCs to vasculogenesis continues after birth. OPCs release hypoxia-inducible factor, which facilitates endothelial cell proliferation and BV formation in the corpus callosum [83] (Fig. 1E). In the mouse model of middle cerebral artery occlusion, transplantation of OPCs promoted functional angiogenesis. Further mechanism investigations showed that OPC-derived Wnt7 acts on β-catenin in endothelial cells, thereby facilitating angiogenesis and improving neurological outcome [78] (Fig. 1E). In addition, the barrier function of the BBB is achieved by the tight junctions formed between endothelial cells. Tumor growth factor β (TGFβ) released from OPCs promotes the expression of tight junction proteins and increases BBB integrity in vitro [61] (Fig. 1E). Specific deletion of TGFβ in OPCs resulted in cerebral hemorrhage and loss of BBB function in the neonatal mice. Please note, during development, endothelial cell-derived TGFβ in turn is crucial for OPC specification from neural progenitor cells [55], suggesting bi-directional communication between endothelial cells and OPCs is crucial for many biological processes. In addition, OPCs may regulate angiogenesis by secreting matrix metallopeptidase (MMP) [62] (Fig. 1E). The extracellular matrix (ECM) is involved in the entire process of angiogenesis, including endothelial cell migration, invasion, proliferation, and survival [47]. Of note, OPCs secrete MMP9 during migration [62]. Further studies are required to demonstrate whether OPC-derived MMP9 regulates angiogenesis.

OPCs interact not only with endothelial cells, but also with astrocytes and pericytes [37, 49, 69], the central elements of the BBB. Required by neuronal activity, astrocytes control cerebral blood flow via their endfeet that wrap the BV [18]. As OPCs migrate along the BV, extensive interaction with astrocytes, particularly with endfeet, is expected. Perivascular OPCs detach from the BV at the site of astroglial endfeet [69] (Fig. 1E). However, when Wnt signaling in OPCs is genetically inhibited, OPC migration is impaired and OPCs form aberrant clusters along the BVs. This abnormal physical occupation results in the loss of astroglial endfeet on the BVs and ultimately an increase in BBB permeability [49]. These studies suggest that an orchestrated interaction between OPCs and astroglial endfeet on the BVs is critical for BBB integrity. OPCs also regulate the pericyte population. Conditioned medium from cultured OPCs promoted pericyte proliferation in vitro, suggesting another pathway by which OPCs regulate BBB function [37].

In summary, OPCs indirectly regulate the neural network activity by interacting with endothelial cells, astrocytes, and pericytes, thereby ultimately mediating BBB function and brain homeostasis.

## Immunomodulation by OPCs

In many neuropathological conditions, such as acute brain injury, multiple sclerosis, and Alzheimer’s disease, there is a significant loss of neurons and disruption of neural circuits. Similar to microglia and astrocytes, OPCs respond to these insults and are involved in disease progression and regeneration. Morphologically, OPCs undergo hypertrophy, with their processes becoming shorter and thicker under pathological conditions [32]. More importantly, OPCs migrate to lesion sites and subsequently become highly proliferative to replace oligodendrocytes lost in case of acute injury or demyelination [25, 80]. These responses contribute to several pathophysiological processes, including glial scar formation and remyelination [60]. Additionally, in pathological contexts including Alzheimer’s disease (AD), depression, and epilepsy, OPCs alter their gene expression profile [42, 45, 54]. For example, in AD pathology, OPCs downregulate Olig1 and Sox8 [42], the genes involved in specification of oligodendrocyte [17, 68], which may explain the myelin deficit in AD [75]. As well, in the OPCs from temporal lobe epilepsy patients, genes related to myelination were downregulated [54]. These changes influence the fate commitment of OPCs in pathological context.

However, recent studies indicate that during neuroinflammation and demyelination conditions, OPCs change their phenotype to modulate response of immune cells. Depletion of OPCs using NG2-HSVtk transgenic mice, where herpes simplex virus thymidine kinase (HSVtk) is expressed under the control of the NG2 promoter, induced hippocampal neuronal death as a result of neuroinflammation triggered by microglial activation [46]. However, application of hepatocyte growth factor, potentially derived from OPCs, was able to reverse the neuronal loss and microglial abnormalities. This observation suggests that OPCs maintain the homeostatic signature of microglia in the healthy CNS. It is important to note that the authors claim that only OPCs were ablated by this strategy, without changing the coverage of pericytes. Although the pericyte coverage is unchanged, but their function, such as regulating blood vessel contraction and blood flow in the brain [50], might have been altered. Hence, there may still be other mechanisms contributing to these changes: (1) neuroinflammation could be caused by the increased BBB permeability due to the loss of perivascular OPCs or dysfunctional pericytes, as mentioned above; (2) it cannot be ruled out that extensive OPC death in the transgenic mice caused microglial activation [46]. Nevertheless, another independent study utilizing NG2-Cre mice crossbred with DTR^fl/fl^ (diphtheria toxin receptor) mice [85] demonstrated that OPCs, but not mature oligodendrocytes (using the PLP-Cre x DTR^fl/fl^ line), are crucial to regulate the microglial response to lipopolysaccharide (LPS)-induced neuroinflammation. In these NG2-Cre x DTR^fl/fl^ mice, about 50% of OPCs were depleted in the absence of LPS challenge, but there was no presence of BBB leakage, suggesting that pericyte function remains unaltered in these mutant mice. Following LPS stimulation, pro-inflammatory cytokines (IL-1β, IL-6, IL-12β, TNF-α, iNOS) showed significant upregulation in the brains of mutant mice compared to controls, suggesting that the loss of OPCs exacerbates microglial responses during neuroinflammation. Through a combination of transcriptomic analysis and co-culture approaches, it was demonstrated that tumor growth factor β (TGFβ) derived from OPCs acts on the TGFβ receptor in microglia, thereby regulating CX3CR1-mediated microglial immune responses [85] (Fig. 1F). Hence, these studies strongly suggest that OPCs may modulate microglial activity in health and disease.

Bi-directional communication between OPCs and other immune cells has also been suggested by many studies [2, 14, 29]. The modulation of T cell responses by OPCs has primarily been studied in the context of multiple sclerosis (MS) (Fig. 1G). In demyelinating lesions, OPCs release chemokines such as C-C motif ligand 2 (CCL2), CCL5 and CXCL10 to recruit T cells [44], and they activate T cells by expressing major histocompatibility complex (MHC) classes I and II, as well as antigens CD273 and CD274 (also known as programmed death ligand (PD-L)2 and PD-L1, respectively) [20, 29, 88] (reviewed by Cabeza-Fernández et al, [14]) (Fig. 1G). When exposed to cerebrospinal fluid (CSF) from MS patients, especially CSF from patients in the phase of progressive MS (pMS), OPCs upregulate PD-L1, which suppresses T cell-induced inflammation [88]. In addition, CSF from pMS patients reduces the expression of MHC-II and TNF-α, as well as the activation of NF-kB in OPCs, compared to CSF from patients in the relapsing phase of MS. Thus, OPCs exposed to pMS CSF hinder T cell activation and proliferation [88]. Previous studies have noted that fewer monocytes are present in the CNS during pMS compared to the rMS phase. Therefore, it is tempting to speculate that OPCs alter their phenotype under different conditions, thereby modulating T cell activity. The conversion of OPCs to a pro-inflammatory phenotype may be mediated by the low-density lipoprotein receptor-related protein (LRP1). OPCs lacking LRP1 express lower levels of MHC-I, MHC-II, and immunoproteasome [3, 23] (Fig. 1G). Interestingly, LRP1 expression is increased in the MS lesion compared to the surrounding healthy tissue [16]. Although it is not clear which phase of MS the patients were in, these studies link the LRP1 to the shift in the OPC phenotype in MS. Taken together, therapeutic strategies should not only focus on the precursor functions of OPCs, but also consider the immunomodulatory roles of OPCs.

## Conclusion

OPCs have been extensively studied for their proliferative and differentiation mechanisms. However, recent studies have revealed a myriad of additional functions performed by OPCs that significantly impact brain function in both healthy and diseased states. Remarkably, OPCs not only receive synaptic input from neurons, but also release neuromodulators that effectively modulate neuronal density, activity, local neural circuits, and synaptic plasticity. In addition, OPCs play a crucial role as key regulators of the blood-brain barrier (BBB), engaging in intensive interactions with other cellular components to ensure proper barrier function. Furthermore, by expressing genes associated with immune cells, OPCs exert phagocytic and immunomodulatory functions, which hold significance in both healthy and various neuropathological conditions. The comprehensive understanding of OPCs’ contribution to neural circuits is of paramount importance for unraveling complex neuropathologies. Ultimately, such insights may pave the way for novel approaches to tackle diseases, particularly those with limited efficacy in neuron-specific treatments.

## Data Availability

Not applicable.

## References

[1] Ahmad AS, Ottallah H, Maciel CB, Strickland M, Doré S (2019). Role of the L-PGDS-PGD2-DP1 receptor axis in sleep regulation and neurologic outcomes. Sleep.

[2] Akay LA, Effenberger AH, Tsai LH (2021). Cell of all trades: oligodendrocyte precursor cells in synaptic, vascular, and immune function. Genes Dev.

[3] Asher RA, Morgenstern DA, Fidler PS, Adcock KH, Oohira A, Braistead JE, Levine JM, Margolis RU, Rogers JH, Fawcett JW (2000). Neurocan is upregulated in injured brain and in cytokine-treated astrocytes. J Neurosci.

[4] Auguste YSS, Ferro A, Kahng JA, Xavier AM, Dixon JR, Vrudhula U, Nichitiu AS, Rosado D, Wee TL, Pedmale UV, Cheadle L (2022). Oligodendrocyte precursor cells engulf synapses during circuit remodeling in mice. Nat Neurosci.

[5] Bai X, Kirchhoff F, Scheller A (2021). Oligodendroglial GABAergic signaling: more than inhibition!. Neurosci Bull.

[6] Balia M, Vélez-Fort M, Passlick S, Schäfer C, Audinat E, Steinhäuser C, Seifert G, Angulo MC (2015). Postnatal down-regulation of the GABAA receptor γ2 subunit in neocortical NG2 cells accompanies synaptic-to-extrasynaptic switch in the GABAergic transmission mode. Cereb Cortex.

[7] Beiter RM, Rivet-Noor C, Merchak AR, Bai R, Johanson DM, Slogar E, Sol-Church K, Overall CC, Gaultier A (2022). Evidence for oligodendrocyte progenitor cell heterogeneity in the adult mouse brain. Sci Rep.

[8] Benamer N, Vidal M, Balia M, Angulo MC (2020). Myelination of parvalbumin interneurons shapes the function of cortical sensory inhibitory circuits. Nat Commun.

[9] Bergles DE, Roberts JD, Somogyi P, Jahr CE (2000). Glutamatergic synapses on oligodendrocyte precursor cells in the hippocampus. Nature.

[10] Birey F, Kloc M, Chavali M, Hussein I, Wilson M, Christoffel DJ, Chen T, Frohman MA, Robinson JK, Russo SJ, Maffei A, Aguirre A (2015). Genetic and stress-induced loss of NG2 glia triggers emergence of depressive-like behaviors through reduced secretion of FGF2. Neuron.

[11] Buchanan J, Elabbady L, Collman F, Jorstad NL, Bakken TE, Ott C, Glatzer J, Bleckert AA, Bodor AL, Brittain D, Bumbarger DJ (2022). Oligodendrocyte precursor cells ingest axons in the mouse neocortex. Proc Natl Acad Sci U S A.

[12] Butt AM, Duncan A, Hornby MF, Kirvell SL, Hunter A, Levine JM, Berry M (1999). Cells expressing the NG2 antigen contact nodes of Ranvier in adult CNS white matter. Glia.

[13] Butt AM, Hamilton N, Hubbard P, Pugh M, Ibrahim M (2005). Synantocytes: the fifth element. J Anat.

[14] Cabeza-Fernández S, White JA, McMurran CE, Gómez-Sánchez JA, de la Fuente AG (2023). Immune-stem cell crosstalk in the central nervous system: how oligodendrocyte progenitor cells interact with immune cells. Immunol Cell Biol.

[15] Cheadle L, Tzeng CP, Kalish BT, Harmin DA, Rivera S, Ling E, Nagy MA, Hrvatin S, Hu L, Stroud H, Burkly LC, Chen C, Greenberg ME (2018). Visual experience-dependent expression of Fn14 is required for retinogeniculate refinement. Neuron.

[16] Chuang TY, Guo Y, Seki SM, Rosen AM, Johanson DM, Mandell JW, Lucchinetti CF, Gaultier A (2016). LRP1 expression in microglia is protective during CNS autoimmunity. Acta Neuropathol Commun.

[17] Dai J, Bercury KK, Ahrendsen JT, Macklin WB (2015). Olig1 function is required for oligodendrocyte differentiation in the mouse brain. J Neurosci.

[18] Daneman R, Prat A (2015). The blood-brain barrier. Cold Spring Harb Perspect Biol.

[19] Daneman R, Zhou L, Kebede AA, Barres BA (2010). Pericytes are required for blood-brain barrier integrity during embryogenesis. Nature.

[20] Falcão AM, van Bruggen D, Marques S, Meijer M, Jäkel S, Agirre E, Samudyata FEM, Vanichkina DP, Ffrench-Constant C, Williams A, Guerreiro-Cacais AO, Castelo-Branco G (2018). Disease-specific oligodendrocyte lineage cells arise in multiple sclerosis. Nat Med.

[21] Fang LP, Liu Q, Meyer E, Welle A, Huang W, Scheller A, Kirchhoff F, Bai X (2022). A subset of OPCs do not express Olig2 during development which can be increased in the adult by brain injuries and complex motor learning. Glia.

[22] Fang LP, Zhao N, Caudal LC, Chang HF, Zhao R, Lin CH, Hainz N, Meier C, Bettler B, Huang W, Scheller A, Kirchhoff F, Bai X (2022). Impaired bidirectional communication between interneurons and oligodendrocyte precursor cells affects social cognitive behavior. Nat Commun.

[23] Fernandez-Castaneda A, Chappell MS, Rosen DA, Seki SM, Beiter RM, Johanson DM, Liskey D, Farber E, Onengut-Gumuscu S, Overall CC, Dupree JL, Gaultier A (2020). The active contribution of OPCs to neuroinflammation is mediated by LRP1. Acta Neuropathol.

[24] Habermacher C, Angulo MC, Benamer N (2019). Glutamate versus GABA in neuron-oligodendroglia communication. Glia.

[25] Hughes EG, Kang SH, Fukaya M, Bergles DE (2013). Oligodendrocyte progenitors balance growth with self-repulsion to achieve homeostasis in the adult brain. Nat Neurosci.

[26] Jung M, Krämer E, Grzenkowski M, Tang K, Blakemore W, Aguzzi A, Khazaie K, Chlichlia K, von Blankenfeld G, Kettenmann H (1995). Lines of murine oligodendroglial precursor cells immortalized by an activated neu tyrosine kinase show distinct degrees of interaction with axons in vitro and in vivo. Eur J Neurosci.

[27] Karadottir R, Cavelier P, Bergersen LH, Attwell D (2005). NMDA receptors are expressed in oligodendrocytes and activated in ischaemia. Nature.

[28] Kessaris N, Fogarty M, Iannarelli P, Grist M, Wegner M, Richardson WD (2006). Competing waves of oligodendrocytes in the forebrain and postnatal elimination of an embryonic lineage. Nat Neurosci.

[29] Kirby L, Jin J, Cardona JG, Smith MD, Martin KA, Wang J, Strasburger H, Herbst L, Alexis M, Karnell J, Davidson T, Dutta R, Goverman J, Bergles D, Calabresi PA (2019). Oligodendrocyte precursor cells present antigen and are cytotoxic targets in inflammatory demyelination. Nat Commun.

[30] Larson VA, Mironova Y, Vanderpool KG, Waisman A, Rash JE, Agarwal A, Bergles DE (2018). Oligodendrocytes control potassium accumulation in white matter and seizure susceptibility. Elife.

[31] Lepiemme F, Stoufflet J, Javier-Torrent M, Mazzucchelli G, Silva CG, Nguyen L (2022). Oligodendrocyte precursors guide interneuron migration by unidirectional contact repulsion. Science.

[32] Levine JM, Reynolds R, Fawcett JW (2001). The oligodendrocyte precursor cell in health and disease. Trends Neurosci.

[33] Lewis S (2011). Development: microglia go pruning. Nat Rev Neurosci.

[34] Lim L, Mi D, Llorca A, Marín O (2018). Development and functional diversification of cortical interneurons. Neuron.

[35] Lin SC, Bergles DE (2004). Synaptic signaling between GABAergic interneurons and oligodendrocyte precursor cells in the hippocampus. Nat Neurosci.

[36] Liu Y, Shen X, Zhang Y, Zheng X, Cepeda C, Wang Y, Duan S, Tong X (2023). Interactions of glial cells with neuronal synapses, from astrocytes to microglia and oligodendrocyte lineage cells. Glia.

[37] Maki T, Maeda M, Uemura M, Lo EK, Terasaki Y, Liang AC, Shindo A, Choi YK, Taguchi A, Matsuyama T, Takahashi R, Ihara M, Arai K (2015). Potential interactions between pericytes and oligodendrocyte precursor cells in perivascular regions of cerebral white matter. Neurosci Lett.

[38] Maldonado PP, Velez-Fort M, Levavasseur F, Angulo MC (2013). Oligodendrocyte precursor cells are accurate sensors of local K+ in mature gray matter. J Neurosci.

[39] Marisca R, Hoche T, Agirre E, Hoodless LJ, Barkey W, Auer F, Castelo-Branco G, Czopka T (2020). Functionally distinct subgroups of oligodendrocyte precursor cells integrate neural activity and execute myelin formation. Nat Neurosci.

[40] Marques S, van Bruggen D, Vanichkina DP, Floriddia EM, Munguba H, Väremo L, Giacomello S, Falcão AM, Meijer M, Björklund Å, Hjerling-Leffler J, Taft RJ, Castelo-Branco G (2018). Transcriptional convergence of oligodendrocyte lineage progenitors during development. Dev Cell.

[41] Marques S, Zeisel A, Codeluppi S, Van Bruggen D, Mendanha Falcão A, Xiao L, Li H, Häring M, Hochgerner H, Romanov RA, Gyllborg D (2016). Oligodendrocyte heterogeneity in the mouse juvenile and adult central nervous system. Science.

[42] Mathys H, Davila-Velderrain J, Peng Z, Gao F, Mohammadi S, Young JZ, Menon M, He L, Abdurrob F, Jiang X, Martorell AJ, Ransohoff RM, Hafler BP, Bennett DA, Kellis M, Tsai LH (2019). Single-cell transcriptomic analysis of Alzheimer’s disease. Nature.

[43] Minocha S, Valloton D, Brunet I, Eichmann A, Hornung JP, Lebrand C (2015). NG2 glia are required for vessel network formation during embryonic development. Elife.

[44] Moyon S, Dubessy AL, Aigrot MS, Trotter M, Huang JK, Dauphinot L, Potier MC, Kerninon C, Melik Parsadaniantz S, Franklin RJ, Lubetzki C (2015). Demyelination causes adult CNS progenitors to revert to an immature state and express immune cues that support their migration. J Neurosci.

[45] Nagy C, Maitra M, Tanti A, Suderman M, Theroux JF, Davoli MA, Perlman K, Yerko V, Wang YC, Tripathy SJ, Pavlidis P, Mechawar N, Ragoussis J, Turecki G (2020). Single-nucleus transcriptomics of the prefrontal cortex in major depressive disorder implicates oligodendrocyte precursor cells and excitatory neurons. Nat Neurosci.

[46] Nakano M, Tamura Y, Yamato M, Kume S, Eguchi A, Takata K, Watanabe Y, Kataoka Y (2017). NG2 glial cells regulate neuroimmunological responses to maintain neuronal function and survival. Sci Rep.

[47] Neve A, Cantatore FP, Maruotti N, Corrado A, Ribatti D (2014). Extracellular matrix modulates angiogenesis in physiological and pathological conditions. Biomed Res Int.

[48] Nishiyama A, Komitova M, Suzuki R, Zhu X (2009). Polydendrocytes (NG2 cells): multifunctional cells with lineage plasticity. Nat Rev Neurosci.

[49] Niu J, Tsai HH, Hoi KK, Huang N, Yu G, Kim K, Baranzini SE, Xiao L, Chan JR, Fancy SPJ (2019). Aberrant oligodendroglial-vascular interactions disrupt the blood-brain barrier, triggering CNS inflammation. Nat Neurosci.

[50] Nortley R, Korte N, Izquierdo P, Hirunpattarasilp C, Mishra A, Jaunmuktane Z, Kyrargyri V, Pfeiffer T, Khennouf L, Madry C, Gong H, Richard-Loendt A, Huang W, Saito T, Saido TC, Brandner S, Sethi H, Attwell D (2019). Amyloid β oligomers constrict human capillaries in Alzheimer’s disease via signaling to pericytes. Science.

[51] Ong WY, Levine JM (1999). A light and electron microscopic study of NG2 chondroitin sulfate proteoglycan-positive oligodendrocyte precursor cells in the normal and kainate-lesioned rat hippocampus. Neuroscience.

[52] Orduz D, Benamer N, Ortolani D, Coppola E, Vigier L, Pierani A, Angulo MC (2019). Developmental cell death regulates lineage-related interneuron-oligodendroglia functional clusters and oligodendrocyte homeostasis. Nat Commun.

[53] Orduz D, Maldonado PP, Balia M, Vélez-Fort M, de Sars V, Yanagawa Y, Emiliani V, Angulo MC (2015). Interneurons and oligodendrocyte progenitors form a structured synaptic network in the developing neocortex. Elife.

[54] Pai B, Tome-Garcia J, Cheng WS, Nudelman G, Beaumont KG, Ghatan S, Panov F, Caballero E, Sarpong K, Marcuse L, Yoo J, Jiang Y, Schaefer A, Akbarian S, Sebra R, Pinto D, Zaslavsky E, Tsankova NM (2022). High-resolution transcriptomics informs glial pathology in human temporal lobe epilepsy. Acta Neuropathol Commun.

[55] Paredes I, Vieira JR, Shah B, Ramunno CF, Dyckow J, Adler H, Richter M, Schermann G, Giannakouri E, Schirmer L, Augustin HG, Ruiz de Almodóvar C (2021). Oligodendrocyte precursor cell specification is regulated by bidirectional neural progenitor-endothelial cell crosstalk. Nat Neurosci.

[56] Ronzano R, Roux T, Thetiot M, Aigrot MS, Richard L, Lejeune FX, Mazuir E, Vallat JM, Lubetzki C, Desmazières A (2021). Microglia-neuron interaction at nodes of Ranvier depends on neuronal activity through potassium release and contributes to remyelination. Nat Commun.

[57] Sakry D, Neitz A, Singh J, Frischknecht R, Marongiu D, Biname F, Perera SS, Endres K, Lutz B, Radyushkin K, Trotter J, Mittmann T (2014). Oligodendrocyte precursor cells modulate the neuronal network by activity-dependent ectodomain cleavage of glial NG2. PLoS Biol.

[58] Sakry D, Yigit H, Dimou L, Trotter J (2015). Oligodendrocyte precursor cells synthesize neuromodulatory factors. PLoS One.

[59] Saper CB, Romanovsky AA, Scammell TE (2012). Neural circuitry engaged by prostaglandins during the sickness syndrome. Nat Neurosci.

[60] Scheller A, Bai X, Kirchhoff F (2017). The role of the oligodendrocyte lineage in acute brain trauma. Neurochem Res.

[61] Seo JH, Maki T, Maeda M, Miyamoto N, Liang AC, Hayakawa K, Pham LD, Suwa F, Taguchi A, Matsuyama T, Ihara M, Kim KW, Lo EH, Arai K (2014). Oligodendrocyte precursor cells support blood-brain barrier integrity via TGF-β signaling. PLoS One.

[62] Seo JH, Miyamoto N, Hayakawa K, Pham LD, Maki T, Ayata C, Kim KW, Lo EH, Arai K (2013). Oligodendrocyte precursors induce early blood-brain barrier opening after white matter injury. J Clin Invest.

[63] Serwanski DR, Jukkola P, Nishiyama A (2017). Heterogeneity of astrocyte and NG2 cell insertion at the node of Ranvier. J Comp Neurol.

[64] Sherafat A, Pfeiffer F, Nishiyama A (2021). Shaping of regional differences in oligodendrocyte dynamics by regional heterogeneity of the pericellular microenvironment. Front Cell Neurosci.

[65] Shimizu T, Mizuno N, Amano T, Hayaishi O (1979). Prostaglandin D2, a neuromodulator. Proc Natl Acad Sci U S A.

[66] Southwell DG, Paredes MF, Galvao RP, Jones DL, Froemke RC, Sebe JY, Alfaro-Cervello C, Tang Y, Garcia-Verdugo JM, Rubenstein JL, Baraban SC, Alvarez-Buylla A (2012). Intrinsically determined cell death of developing cortical interneurons. Nature.

[67] Spitzer SO, Sitnikov S, Kamen Y, Evans KA, Kronenberg-Versteeg D, Dietmann S, de Faria O, Agathou S, Káradóttir RT (2019). Oligodendrocyte progenitor cells become regionally diverse and heterogeneous with age. Neuron.

[68] Stolt CC, Schmitt S, Lommes P, Sock E, Wegner M (2005). Impact of transcription factor Sox8 on oligodendrocyte specification in the mouse embryonic spinal cord. Dev Biol.

[69] Su Y, Wang X, Yang Y, Chen L, Xia W, Hoi KK, Li H, Wang Q, Yu G, Chen X, Wang S, Wang Y, Xiao L, Verkhratsky A, Fancy SPJ, Yi C, Niu J (2022). Astrocyte endfoot formation controls the termination of oligodendrocyte precursor cell perivascular migration during development. Neuron.

[70] Sun W, Matthews EA, Nicolas V, Schoch S, Dietrich D (2016). NG2 glial cells integrate synaptic input in global and dendritic calcium signals. Elife.

[71] Tanti A, Belliveau C, Nagy C, Maitra M, Denux F, Perlman K, Chen F, Mpai R, Canonne C, Théberge S, McFarquhar A, Davoli MA, Belzung C, Turecki G, Mechawar N (2022). Child abuse associates with increased recruitment of perineuronal nets in the ventromedial prefrontal cortex: a possible implication of oligodendrocyte progenitor cells. Mol Psychiatry.

[72] Timmermann A, Tascio D, Jabs R, Boehlen A, Domingos C, Skubal M, Huang W, Kirchhoff F, Henneberger C, Bilkei-Gorzo A, Seifert G, Steinhäuser C (2023). Dysfunction of NG2 glial cells affects neuronal plasticity and behavior. Glia.

[73] Trotter J, Karram K, Nishiyama A (2010). NG2 cells: Properties, progeny and origin. Brain Res Rev.

[74] Tsai HH, Niu J, Munji R, Davalos D, Chang J, Zhang H, Tien AC, Kuo CJ, Chan JR, Daneman R, Fancy SP (2016). Oligodendrocyte precursors migrate along vasculature in the developing nervous system. Science.

[75] Vanzulli I, Papanikolaou M, De-La-Rocha IC, Pieropan F, Rivera AD, Gomez-Nicola D, Verkhratsky A, Rodríguez JJ, Butt AM (2020). Disruption of oligodendrocyte progenitor cells is an early sign of pathology in the triple transgenic mouse model of Alzheimer’s disease. Neurobiol Aging.

[76] Vigano F, Mobius W, Gotz M, Dimou L (2013). Transplantation reveals regional differences in oligodendrocyte differentiation in the adult brain. Nat Neurosci.

[77] Walz W (2000). Role of astrocytes in the clearance of excess extracellular potassium. Neurochem Int.

[78] Wang LP, Pan J, Li Y, Geng J, Liu C, Zhang LY, Zhou P, Tang YH, Wang Y, Zhang Z, Yang GY (2022). Oligodendrocyte precursor cell transplantation promotes angiogenesis and remyelination via Wnt/. J Cereb Blood Flow Metab.

[79] Wang Y, Su Y, Yu G, Wang X, Chen X, Yu B, Cheng Y, Li R, Sáez JC, Yi C, Xiao L, Niu J (2021). Reduced oligodendrocyte precursor cell impairs astrocytic development in early life stress. Adv Sci (Weinh).

[80] Wegener A, Deboux C, Bachelin C, Frah M, Kerninon C, Seilhean D, Weider M, Wegner M, Nait-Oumesmar B (2015). Gain of Olig2 function in oligodendrocyte progenitors promotes remyelination. Brain.

[81] Xiao Y, Petrucco L, Hoodless LJ, Portugues R, Czopka T (2022). Oligodendrocyte precursor cells sculpt the visual system by regulating axonal remodeling. Nat Neurosci.

[82] Yu G, Su Y, Guo C, Yi C, Yu B, Chen H, Cui Y, Wang X, Wang Y, Chen X, Wang S, Wang Q, Chen X, Hu X, Mei F, Verkhratsky A, Xiao L, Niu J (2022). Pathological oligodendrocyte precursor cells revealed in human schizophrenic brains and trigger schizophrenia-like behaviors and synaptic defects in genetic animal model. Mol Psychiatry.

[83] Yuen TJ, Silbereis JC, Griveau A, Chang SM, Daneman R, Fancy SPJ, Zahed H, Maltepe E, Rowitch DH (2014). Oligodendrocyte-encoded HIF function couples postnatal myelination and white matter angiogenesis. Cell.

[84] Zhang K, Chen S, Yang Q, Guo S, Chen Q, Liu Z, Li L, Jiang M, Li H, Hu J, Pan X, Deng W, Xiao N, Wang B, Wang ZX, Zhang L, Mo W (2022). The oligodendrocyte transcription factor 2 OLIG2 regulates transcriptional repression during myelinogenesis in rodents. Nat Commun.

[85] Zhang SZ, Wang QQ, Yang QQ, Gu HY, Yin YQ, Li YD, Hou JC, Chen R, Sun QQ, Sun YF, Hu G, Zhou JW (2019). NG2 glia regulate brain innate immunity via TGF-β2/TGFBR2 axis. BMC Med.

[86] Zhang X, Liu Y, Hong X, Li X, Meshul CK, Moore C, Yang Y, Han Y, Li WG, Qi X, Lou H, Duan S, Xu TL, Tong X (2021). NG2 glia-derived GABA release tunes inhibitory synapses and contributes to stress-induced anxiety. Nat Commun.

[87] Zhao N, Huang W, Catalin B, Scheller A, Kirchhoff F (2021). L-Type Ca(2+) channels of NG2 glia determine proliferation and NMDA receptor-dependent plasticity. Front Cell Dev Biol.

[88] Zveik O, Fainstein N, Rechtman A, Haham N, Ganz T, Lavon I, Brill L, Vaknin-Dembinsky A (2022). Cerebrospinal fluid of progressive multiple sclerosis patients reduces differentiation and immune functions of oligodendrocyte progenitor cells. Glia.

